# A Micro-Fabricated Force Sensor Using an All Thin Film Piezoelectric Active Sensor

**DOI:** 10.3390/s141222199

**Published:** 2014-11-25

**Authors:** Junwoo Lee, Wook Choi, Yong Kyoung Yoo, Kyo Seon Hwang, Sang-Myung Lee, Sungchul Kang, Jinseok Kim, Jeong Hoon Lee

**Affiliations:** 1 Department of Electrical Engineering, Kwangwoon University, 447-1, Wolgye, Nowon, Seoul 139-701, Korea; E-Mails: mindsjw@gmail.com (J.L.); duddnjsi2@naver.com (W.C.); yongkyoung0108@gmail.com (Y.K.Y.); 2 Korea Institute of Science and Technology (KIST), Seoul 136-791, Korea; E-Mails: kshwang@kist.re.kr (K.S.H.); kasch@kist.re.kr (S.K.); 3 Department of Chemical Engineering, Kangwon National University, Chuncheon 200-701, Korea; E-Mail: sangmyung@kangwon.ac.kr

**Keywords:** force sensor, tactile sensor, piezoelectric, thin film, MEMS

## Abstract

The ability to measure pressure and force is essential in biomedical applications such as minimally invasive surgery (MIS) and palpation for detecting cancer cysts. Here, we report a force sensor for measuring a shear and normal force by combining an arrayed piezoelectric sensors layer with a precut glass top plate connected by four stress concentrating legs. We designed and fabricated a thin film piezoelectric force sensor and proposed an enhanced sensing tool to be used for analyzing gentle touches without the external voltage source used in FET sensors. Both the linear sensor response from 3 kPa to 30 kPa and the exact signal responses from the moving direction illustrate the strong feasibility of the described thin film miniaturized piezoelectric force sensor.

## 1. Introduction

Tactile and force sensing by mimicking human skin has recently received a great deal of attention and much progress has been made in developing highly sensitive force and tactile sensors [1–5]. By emulating human skin, an attempt has been made to develop electronic skin based on a large area of arrays with flexible properties [4,6,7]. Meanwhile, by using a tactile sensor, especially laparoscopic forceps applications with haptic technology, only the direction and quantity of the force need to be measured. Therefore, the tactile sensor does not need to have flexible properties. Tactile sensors can be used for bio-medical applications such as detecting cancer cysts [8]; however, the major hurdle in palpation is the lack of the sensation of touch; surgeons therefore experience difficulty discerning the pathology of the tissue when operating using minimally invasive surgery (MIS) [9]. More specifically, for keyhole surgery, surgeons need to feel the biological structure on which they are operating [10]; however, a sensor system with a high sensitive normal/shear force sensor with a miniaturized system has not yet been demonstrated. Since force sensing can be utilized in both medical surgery equipment and cancer detection, a tactile system with a highly sensitive device is urgently needed for real biomedical applications.

In order for the tactile force sensing system to measure normal compressive stress and horizontal shear stress, Lu and co-workers presented an interfacial stress sensor [11]. Chuang and co-workers designed a tactile sensor for the application of the laparoscope and showed the ability to discriminate the *in vitro* measurements of five different animal tissues [12]. Thanh-Vinh and co-workers proposed tactile sensors with the structure of a PDMS cap and piezoresistive cantilevers with a cavity [13]. Principles and advanced research have been reviewed recently in various literatures [5,7], and several transduction techniques such as piezoresistive, capacitive, piezoelectric and optical methods have been studied for use in tactile sensing materials [7]. For piezoelectric tactile sensors, several materials have been suggested as candidates, such as polyvinylidene fluoride (PVDF), polycrystalline PZT, and ZnO. Among the piezoelectric materials, PVDF is known to be a low-cost polymer material that is applicable for flexible piezoelectrics, however, it does not show better piezoelectric properties than PZT materials [14,15]. By using PZT materials, we suggested a pressure sensor with enhanced sensitivity as a result of integrating a PZT layer with a microstructured polydimethylsiloxane layer. However, the measurements were limited to single points, not an array [16].

Here, we propose the design and fabrication of a force sensor array, for application in laparoscopic forceps, by combining a piezoelectric PZT sensor layer with a rigid glass plate. The piezoelectric sensor layer and the top glass plate were connected with four stress concentrating legs. We designed a piezoelectric transducer with an elastic component rather than viscoelastic materials, since we intended to prevent positional uncertainty in locating contact points and hysteresis caused by viscoelastic behavior. By utilizing piezoelectric (PZT) thin film arrays, we fabricated four piezoelectric sensor arrays and acquired a direct piezoelectric signal from the four sensors without an external operating voltage. Also, we presented static force measurements as well as dynamic force measurements, showing excellent sensitivity and linearity.

## 2. Experimental Section

**Figure 1.**
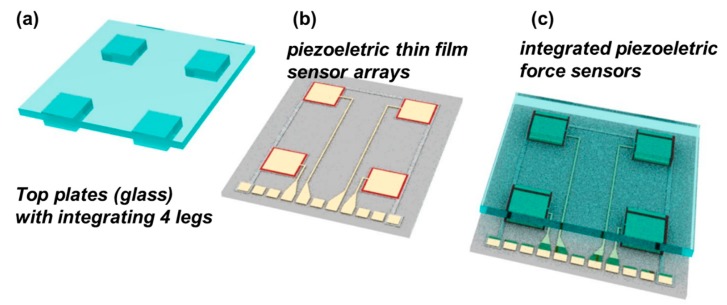
Concept of triaxial force sensor using piezoelectric active sensor arrays.

The basic concept and structure of this microelectromechanical systems (MEMS)-based piezoelectric force sensor are shown in Figure 1. First, the top plates with glass materials are prepared as shown in Figure 1. The top plate contains four prepared glass blocks acting as force concentrating legs. The dimension of each prepared glass block (legs) was 1.8 mm (width) × 1.8 mm (length) with 500 μm thickness. Second, the piezoelectric thin film sensor layer was fabricated on a silicon wafer. We prepared Pb(Zr_0.52_Ti_0.48_)O_3_ thin film using the sol-gel method based on 1,3-propanediol (Sigma-Aldrich, Shanghai, China). A fully detailed process can be found in previous reports [17]. We then fabricated the piezoelectric capacitor using a standard microfabrication method. Third, the top plates with the four legs were integrated with the piezoelectric thin film sensor layer. The top and bottom sensors are simply bonded by spin coated 1 μm thick PDMS adhesive layer. Figure 1c shows the final design comprising the piezoelectric sensor integrated with the top plate and four legs. We designed the force sensor by integrating a thin-film piezoelectric layer fabricated on a silicon wafer with a pre-cut glass top plate. The design mimics the Wii balance board designed by Nintendo. Our force sensor is composed of four pressure sensors with piezoelectric thin film at the corner of the devices. When the pressure was applied to the top glass plates, the location and force direction could be monitored by measuring the signal from the four piezoelectric pressure sensor unit.

**Figure 2.**
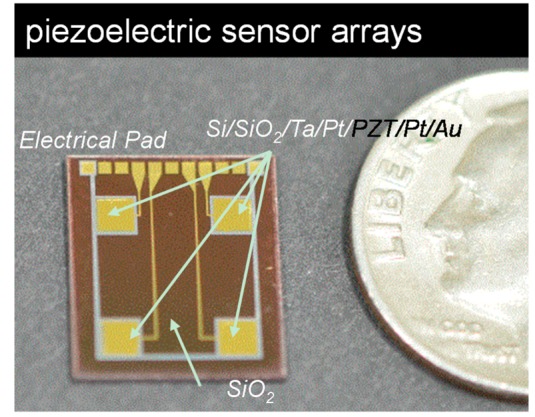
Optical images of piezoelectric sensor unit. The sensors consisted of Si/Ta/Pt/PZT/Pt/Au, and the surface was passivated using a SiO_2_ layer. A US 10 cent coin (17.9 mm in diameter) is shown as a reference for the sensor size.

As the first step of device fabrication, we developed a MEMS thin-film piezoelectric sensor with the dimensions of 10 mm × 12 mm and composed of seven multi-layers, specifically SiO_2_ (100 nm)/Ta (30 nm)/Pt (150 nm)/PZT (1.0 μm)/Pt (100 nm)/SiO_2_ (100 nm)/Au (150 nm), on a 4-inch silicon wafer to function as a pressure sensor as shown in Figure 2. The total thickness of all the piezoelectric functional films was 1.63 μm. The bottom electrode was commonly connected through four force sensor units and the PZT film located with the isolated structure. The prepared Si/SiO_2_/Ta/Pt substrate was annealed at 650 °C for 30 min and PZT (52/48) films were prepared using the sol-gel method with lead acetate trihydrate, zirconium propoxide, titanium isopropoxide, 1,3-propanediol, and acetylacetone as solvents. The films were deposited by spin coating at 3000 rpm for 30 s, subsequently fired at 400 °C for 5 min, and finally annealed at 650 °C. After the thin film deposition, we etched the top Pt, the PZT, and the bottom Pt, sequentially, and then deposited silicon dioxide using plasma-enhanced chemical vapor deposition (PECVD) as shown in previous reports [18–20]. The PZT layer was wet-etched using an etching solution of H_2_O, HCl, and HF at a ratio of 270:15:1. After the fabrication of the via-hole through the silicon dioxide layer located on the top electrode, we deposited Au thin film using a lift-off process. The Au layer was used for the electrical pad which was connected between the top electrodes and the electrical pad. Also, the common bottom electrode was connected with an electrical pad located at the side of the devices. The poling was done under an electric field of 100 kV/cm at 150 °C. The dimensions of the piezoelectric sensor were 10 mm × 12 mm and the dimension of each of the four pressure sensing unit on the piezoelectric sensors was 1.8 mm × 1.8 mm.

## 3. Results and Discussion

**Figure 3.**
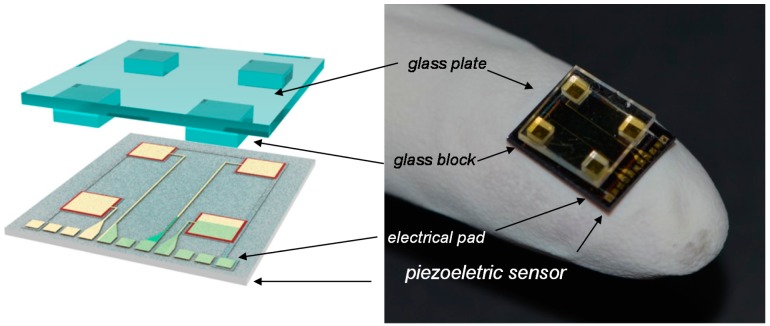
Integration of the four force balances. The glass plate was prepared with four legs in direct contact with the piezo-arrays.

The fully integrated sensors are shown in Figure 3. The dimensions and thickness of the top plate were 9 mm × 9 mm and 500 μm, respectively. When the object contacted any part of the glass plates, the pressure was delivered to the four piezoelectric pressure sensor unit and consequently the piezoelectric signal increased proportional to the applied loads [13].

**Figure 4.**
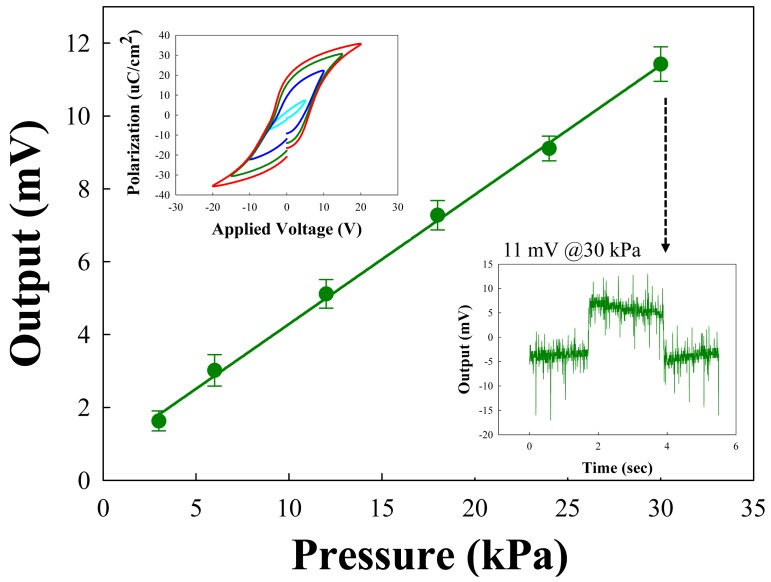
Electrical output with applied pressure on each piezo-sensor unit. The inset shows the P-E curve of the fabricated piezoelectric force sensor.

The concentrated load was focused on the four legs and the forces then generated the piezoelectric signal by direct piezoelectric effects. The static sensor responses with applied forces are shown in Figure 4. In order to apply the forces while simultaneously measuring the electrical signal, we used a digital force gauge (DS2-5N, IMADA, Aichi, Japan) and Labview software to control the applied forces. Also, we used the measurement gadget for multichannel force sensor measurements. The measurement unit consisted of electrical lead outs with nine pins, and the unit was connected with a Labview-controlled PC to measure the electrical signal from the piezoelectric sensor layer (see Supplementary Figure S1). The measurements were performed with each single pressure sensor unit. We applied the forces using a square tip (3.24 mm^2^) at the end of a force gauge and the output voltage was monitored through data acquisition (DAQ) board and Labview software.

The lower-right inset shows that the time sequential electrical output was 11 mV at less than 30 kPa pressure. We applied the pressure for 2 s and measured the electrical output under six different pressure conditions. The pressure range converted from the applied force was calculated as a range from 3 kPa to 30 kPa. A gentle touch, reportedly, refers to a pressure below 10 kPa [2], which is included in the pressure range we measured. For the discrete run, we acquired a linear sensor response from 3 kPa to 30 kPa pressure ranges and the output voltage was linearly proportional to the applied pressures from 1.8 mV to 11 mV. The upper-left inset shows the P-E hysteresis of PZT films, showing good ferroelectric properties.

**Figure 5.**
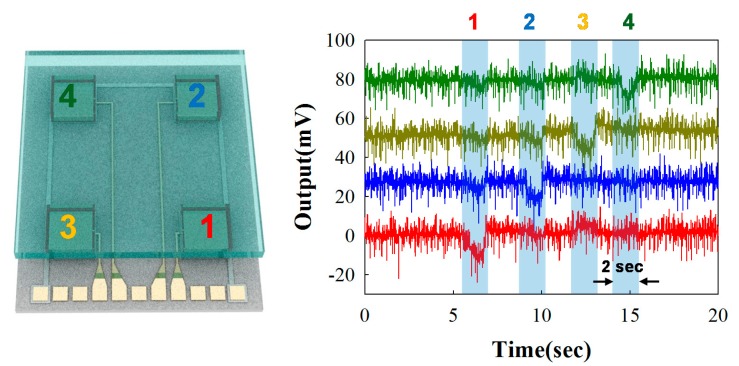
Electrical output signal under normal forces via static pressure with 30 kPa on each sensor unit.

Figure 5 shows the experimental results of the output voltages at less than 30 kPa static force loading on each pressure sensor unit. When the pressure was loaded on the top surface of the glass plate on the pressure sensor 1 (red), the electrical output was detected, while the electrical signal from other sensor units (pressure sensor 2–4) shows no significant output. With a similar pattern, we pushed the top surface area of sensor 2 (blue), and the piezoelectric signal exactly responded to the pushing area and applied pressure. Interestingly, all the signals produced approximately 11 mV, when we applied static force with 30 kPa from pressure sensor units 1 to 4, showing the ability of the arrayed force sensors with good uniformity and reproducibility.

**Figure 6.**
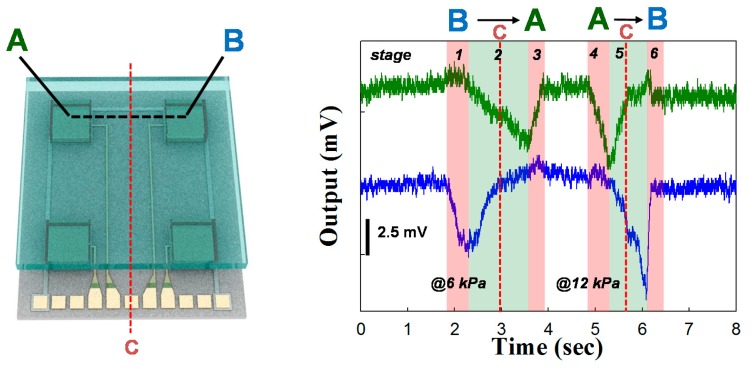
Tangential force electrical output signal when the objects sweep between the A and B sensor units.

To measure the dynamic force loading, we carried out the sweeping of objects under a gentle touch as shown in Figure 6. First, we pushed the objects on the center point of pressure unit B (stage 1), and moved the objects from the B to the A sites under the same forces and speeds (stage 2), then removed the objects when the pushing objects were located at the center of pressure unit A (stage 3). For the sweeping objects, we used ball-pen tips as pushing objects. We applied and moved the gentle touch object with 0.27 cm/s under 5 kPa pressure when dragging objects from B to A, while we increased the speed at 0.44 cm/s under 10 kPa when dragging objects from A to B.

To calculate the applied pressure using ball-pen tips, we first measured two forces using a commercial force gauge (DS2-5N, IMADA) by simply sweeping B to A very gently, and then A to B gently. The measurements were performed 10 times, and the read-out signal from a commercial force gauge was averaged. The averaged pressure by force gauge from the very gentle and gentle sweeping was calculated as 6 kPa and 12 kPa, respectively. Since the gentle touch was defined as the forces of <10 kPa, the forces with the ball-pen tips are in the category of gentle touch. When we contacted the object on the center of pressure sensor B, and then applied the pressure up to 6 kPa, the electrical signal was generated only from pressure sensor unit B and maximized (stage 1 in Figure 6). When the objects moved and located at C point (red dot line in Figure 6), we observed that the signal from unit B decreased and the response from unit A started to increase proportionally to the decreasing amount of signal B (stage 2 in Figure 6). When the objects moved away from point C the signal from A was dominantly produced piezoelectric signal, while that from B was returned to its original value (stage 2 in Figure 6). Finally, when the object reached the center of site A, we removed the forces, and the electric signal from A consequently also returned to its initial status (stage 3 in Figure 6). To check the signal response with reverse direction sweep, we moved the objects from site A to site B and observed the signal response with three stages, consisting of stages 4–6. Since we used the forces with 10 kPa, we acquired the electrical signal which was proportional to the applied pressure. With such a gentle touch, the sensor array produces 3.7 mV and 6 mV with 6 kPa and 12 kPa, respectively. The relaxations with two different gentle sweeps corresponded with the removal of the applied force, demonstrating sufficient speed for practical applications.

## 4. Conclusions/Outlook

In conclusion, we have designed and fabricated a force sensor containing piezoelectric thin films. By mimicking the Wii balance board, we designed a force sensor composed of four pressure sensors with piezoelectric thin film. By measuring the electrical signal from static force loading, we acquired the linear sensor response in a wide dynamic pressure range from 3 kPa to 30 kPa, which is comparable to a very gentle touch. We also observed the ability of the detection of moving direction and forces and could differentiate the force direction by reading the electrical signal as well as the slopes of the signal. The proposed sensor illustrates the strong feasibility of the thin film MEMS piezoelectric sensor array integrated with pre-cut glass plates; this arrayed piezoelectric sensor platform could potentially provide the triaxial force measurements for biomedical tactile application without the need for an external voltage source.

## Supplementary Material
