# Supplementary material for: A Micro-Fabricated Force Sensor Using an All Thin Film Piezoelectric Active Sensor
Source: Sensors (Basel). 2014 Nov 25;14(12):22199–207. doi: 10.3390/s141222199 (PMC4299009; doi:10.3390/s141222199)
Supplement: Supplementary file 1 [file sensors-14-22199-s001.pdf]

OPEN ACCESS

sensors

ISSN 1424-8220

www.mdpi.com/journal/sensors

Supplementary Information

A Micro-Fabricated Force Sensor Using an All Thin Film Piezoelectric Active Sensor. *Sensors* 2014, *14*, 22199-22207

Junwoo Lee [1], Wook Choi [1], Yong Kyoung Yoo [1], Kyo Seon Hwang [2], Sang-Myung Lee [3], Sungchul Kang [2], Jinseok Kim [2,†,*] and Jeong Hoon Lee [1,†,*]

[1] Department of Electrical Engineering, Kwangwoon University, 447-1, Wolgye, Nowon, Seoul 139-701, Korea; E-Mails: mindsjw@gmail.com (J.L.); duddnjsi2@naver.com (W.C.); yongkyoung0108@gmail.com (Y.K.Y.)

[2] Korea Institute of Science and Technology (KIST), Seoul 136-791, Korea; E-Mails: kshwang@kist.re.kr (K.S.H.); kasch@kist.re.kr (S.K.)

[3] Department of Chemical Engineering, Kangwon National University, Chuncheon 200-701, Korea; E-Mail: sangmyung@kangwon.ac.kr

[†] These authors contributed equally to this work.

* Authors to whom correspondence should be addressed; E-Mails: jinseok@kist.re.kr (J.K.); jhlee@kw.ac.kr (J.H.L.); Tel.: +82-2-958-6745 (J.K.); Tel.: +82-2-940-8372 (J.H.L).

Figure S1. Experimental set-up for micro-fabricated piezoelectric force sensor.

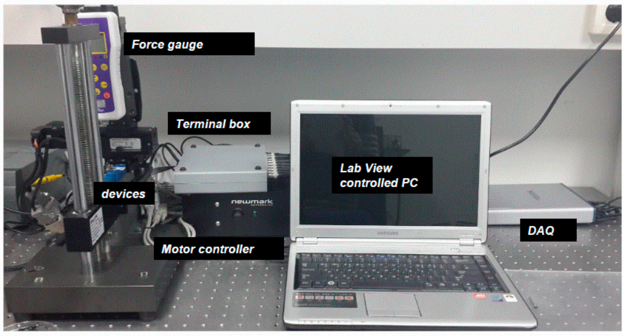

© 2014 by the authors; licensee Molecular Diversity Preservation International, Basel, Switzerland. This article is an open-access article distributed under the terms and conditions of the Creative Commons Attribution license (http://creativecommons.org/licenses/by/4.0/).
